# Open Cystolithotomy Under Local Anesthesia in a High‐Risk Patient With a Giant Bladder Stone and Unilateral Lung Collapse: A Case Report

**DOI:** 10.1155/criu/9312974

**Published:** 2026-10-06

**Authors:** Milan Gyawali, Ritamvara Oli, Sunil Regmi, Purushottam Parajuli, Akash Chitrakar, Rabindra Tamang

**Affiliations:** ^1^ Department of Urology Koshi Hospital Biratnagar Nepal; ^2^ Department of Pulmonology Bir Hospital Kathmandu Nepal

**Keywords:** bladder stone, collapsed lung, high-risk patient, local anesthesia, open cystolithotomy

## Abstract

**Introduction:**

Giant bladder stone is rare in current scenarios. When patients also have severe pulmonary disease, anesthesia becomes high risk. This case illustrates how open cystolithotomy under local anesthesia can be a viable option in such medically compromised patients.

**Case Presentation:**

A 60‐year‐old male from rural Eastern Nepal presented after prolonged history of lower urinary tract symptoms, hematuria, and progressive shortness of breath. Chest imaging revealed a collapsed left lung. Abdominal imaging showed a giant bladder stone (11 ^∗^8 cm). Given the patient′s severely reduced pulmonary reserve, he underwent open cystolithotomy under local anesthesia with mild sedation. The stone was removed successfully with no major perioperative complications.

**Discussion:**

In patients with a collapsed left lung, the anesthetic risk associated with general or spinal anesthesia is significantly high. In such circumstances, local anesthesia with sedation, although uncommon for open bladder surgery, may be considered when the patient′s clinical condition mandates it. Adequate preoperative optimization, including chest physiotherapy and careful perioperative monitoring, is essential. Management requires balancing the risks of anesthesia against the risks of leaving the stone untreated, such as infection, renal damage, and bladder dysfunction.

**Conclusion:**

Open cystolithotomy under local anesthesia with mild sedation may be a feasible alternative in high‐risk cases with giant bladder stone where general/regional anesthesia is contraindicated. Early detection, multidisciplinary management, and resource allocation are essential especially in rural and under‐resourced regions for prevention and management of these cases.

## 1. Introduction

Bladder stones comprise about 5% of all urolithiasis and are more commonly encountered in underdeveloped countries with limited access to healthcare [1, 2]. Bladder stones are mostly secondary in origin, resulting from conditions such as benign prostatic hyperplasia, urethral stricture disease, neurogenic bladder, chronic urinary tract infection, or the presence of foreign bodies within the bladder [1]. Large vesical calculi are rare and typically present in settings of prolonged neglect and poor health‐seeking behavior. It is often associated with significant lower urinary tract symptoms, recurrent urinary tract infections, hematuria, hydronephrosis, and renal impairment [3, 4].

The standard management of giant bladder stones is open cystolithotomy, which allows complete stone removal with minimal risk of residual fragments [1, 5]. Conventionally, this procedure is performed under general or spinal anesthesia. Patients with severe cardiopulmonary comorbidities may not tolerate general or regional anesthesia, posing a significant therapeutic challenge [5]. In such high‐risk cases, alternative anesthetic strategies must be considered. The use of local anesthesia for open cystolithotomy is exceedingly uncommon and sparsely reported in the literature.

We report a rare case of a giant bladder stone in a neglected elderly male with a collapsed left lung, managed successfully by open cystolithotomy under local anesthesia with sedation at a government hospital in Eastern Nepal. This case adds to existing literature by demonstrating the feasibility of open cystolithotomy under local anesthesia in a patient with unilateral lung collapse and large stone burden in a resource‐limited setting. This case report has been prepared in accordance with CARE guidelines.

## 2. Case Presentation

A 60‐year‐old male monk from a remote village in eastern Nepal presented to the urology outpatient department with gradually worsening dysuria, urinary frequency, urgency, suprapubic discomfort, and intermittent gross hematuria for approximately 3 years. He also reported progressive weakness, weight loss, and a chronic productive cough over the preceding few months. The patient lived alone in impoverished conditions at a local temple and had no available past medical records. He was a chronic smoker (> 25 pack‐years) with poor nutritional status and personal hygiene.

On physical examination, the patient appeared cachectic, dehydrated, and tachypneic, with an oxygen saturation of 88% on room air. Abdominal examination revealed suprapubic tenderness with a palpable hard mass. Respiratory examination showed reduced breath sounds with dullness to percussion over the left hemithorax.

Laboratory investigations revealed a hemoglobin level of 10.2 g/dL, a white blood cell count of 13,600/mm^3^, and a serum creatinine level of 1.9 mg/dL. Urinalysis demonstrated pyuria and bacteriuria, and urine culture grew *Escherichia coli*.

Chest radiography demonstrates findings suggestive of a collapsed left lung (Figure 1). Ultrasonography of the abdomen and pelvis revealed a giant bladder stone measuring 11 ^∗^8 cm, a right partial staghorn calculus, bilateral moderate hydroureteronephrosis, and a prostate volume of 30 g. A plain x‐ray of the kidneys, ureters, and bladder (KUB) showed a large radio‐opaque shadow in the pelvic region/prostatic fossa consistent with a giant bladder stone, as well as a radio‐opaque shadow in the right renal fossa suggestive of a renal calculus (Figure 2).

**Figure 1:**
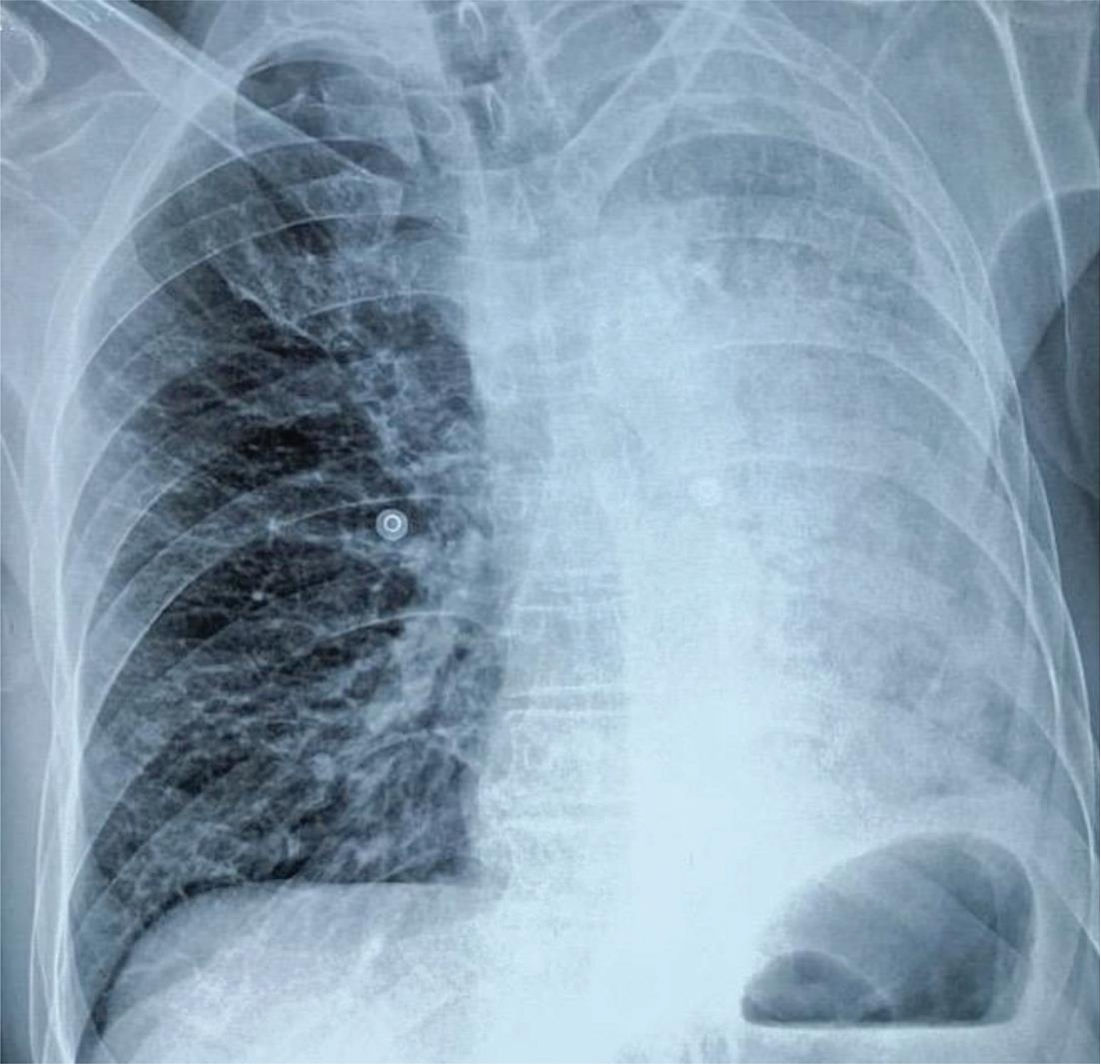
Chest x‐ray demonstrating features suggestive of unilateral left lung collapse.

**Figure 2:**
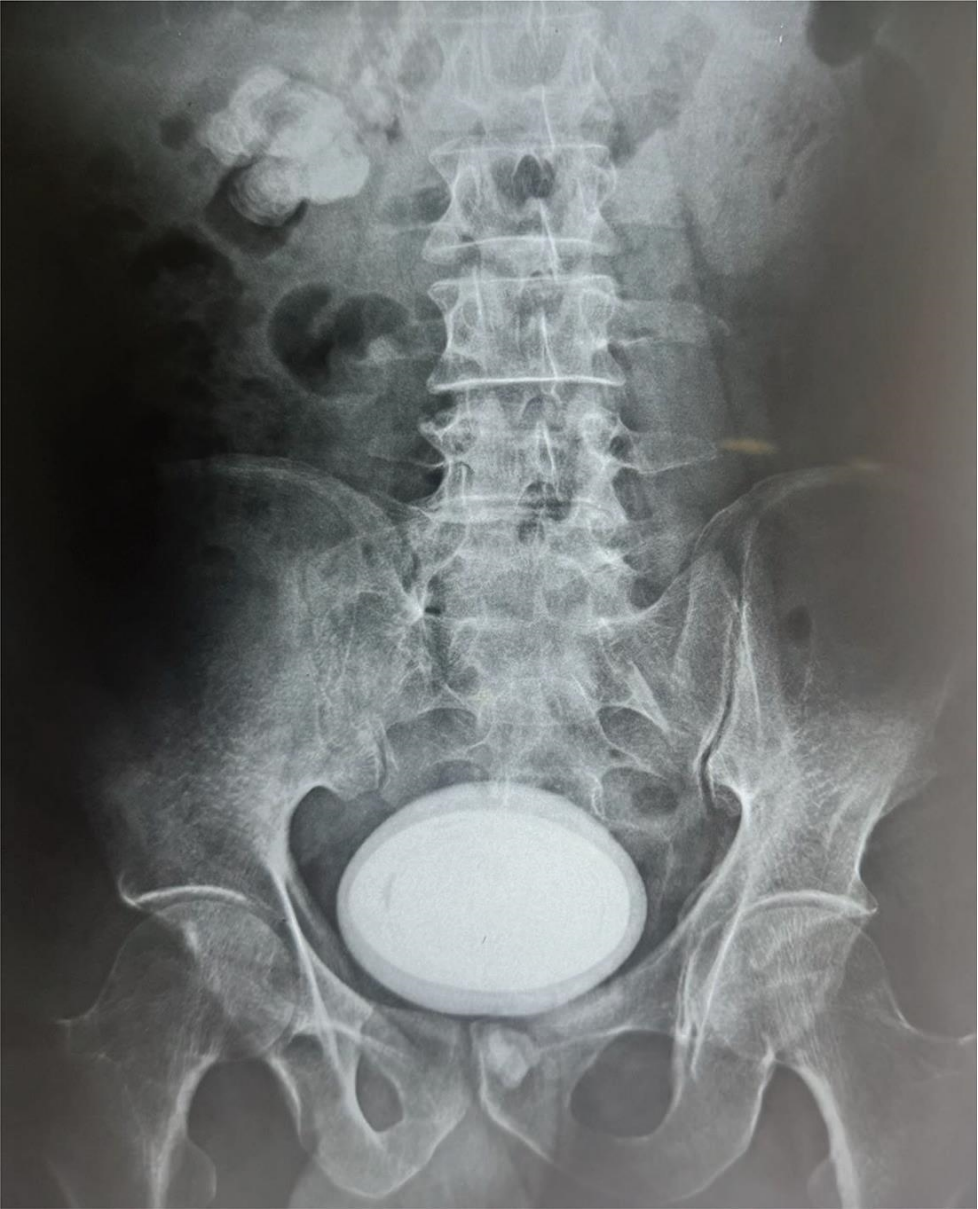
X‐ray KUB showing large bladder stone projecting over the prostatic fossa and right‐sided partial staghorn calculus.

Given the patient′s severely compromised pulmonary reserve and high anesthetic risk (American Society of Anesthesiologists‐IV), a multidisciplinary consultation involving internal medicine and anesthesia was conducted. The consensus was to proceed with open cystolithotomy under local anesthesia with sedation after preoperative optimization with intravenous antibiotics and pulmonary rehabilitation for a period of 7 days prior to surgery.

Local anesthesia was infiltrated with a mixture of 1% lignocaine (30 mL, 10 mg/mL) and 0.25% bupivacaine (20 mL, 2.5 mg/mL) and for conscious sedation, midazolam (1 mg) and fentanyl (50 *μ*g) which were titrated to achieve a Ramsay sedation score of 3. The patient was under continuous monitoring by an anesthesiologist using pulse oximetry, noninvasive blood pressure, and electrocardiography. A 12‐cm suprapubic Pfannenstiel incision was made, followed by layer‐by‐layer infiltration of local anesthetic into the abdominal wall and perivesical fat. The bladder was opened longitudinally. A large stone measuring 10 ^∗^8 cm was found adherent to the bladder mucosa and was carefully separated and removed (Figure 3). Two smaller stones located in the prostatic fossa were also extracted. The bladder mucosa was thoroughly inspected for any suspicious lesions and was subsequently closed in two layers using 2‐0 Vicryl sutures. A per‐urethral Foley catheter and a 20‐Fr perivesical drain were placed, and the abdominal wall was closed in layers. The procedure was well tolerated, with adequate pain control throughout. The patient remained hemodynamically stable, with no episodes of desaturation or respiratory compromise during surgery.

**Figure 3:**
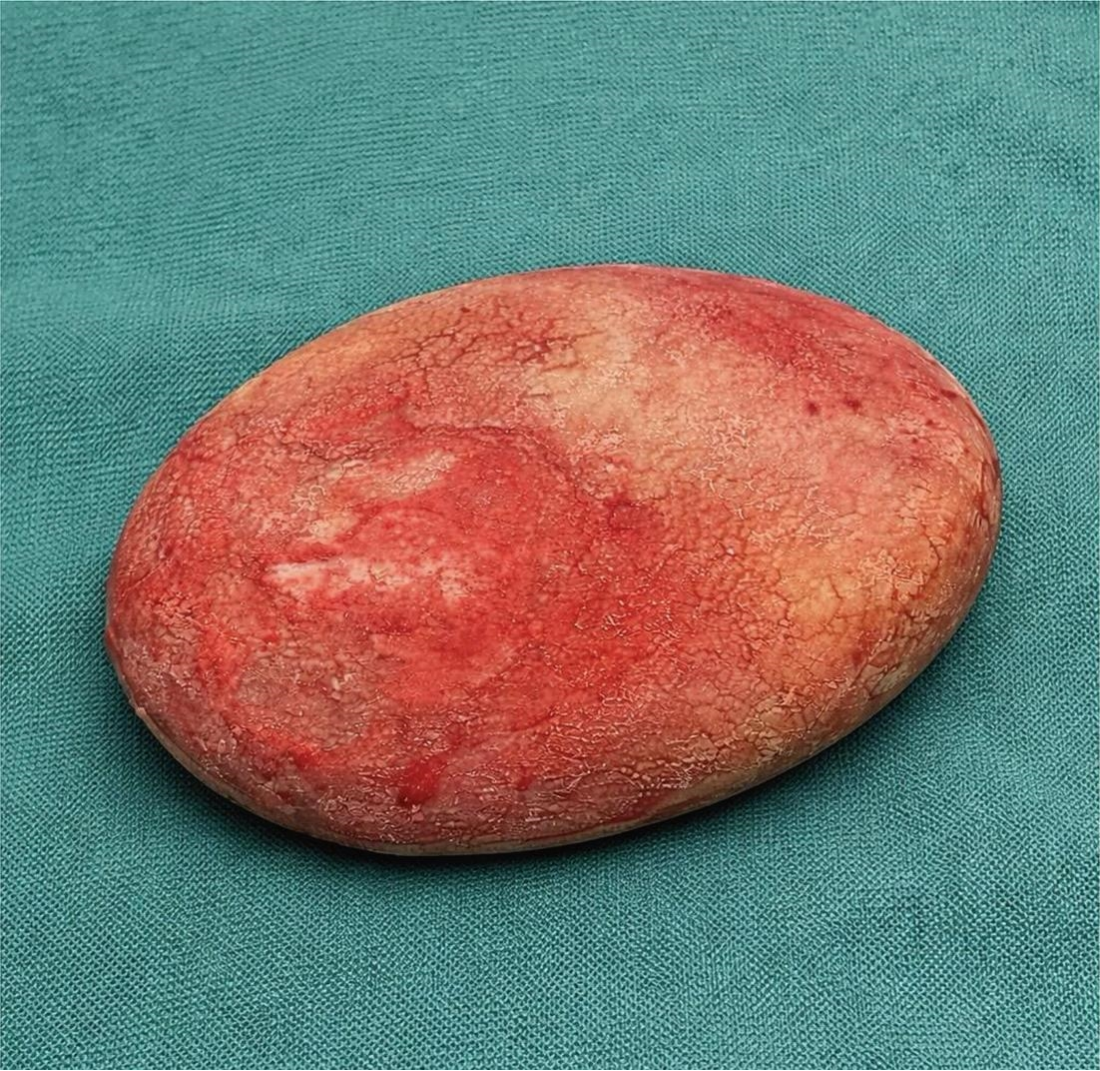
Postoperative specimen of large bladder stone measuring 10 ^∗^8 cm.

Postoperatively, the patient was maintained on supplemental oxygen and chest physiotherapy. Intravenous antibiotics were continued for 5 days according to culture sensitivity results. The perivesical drain was removed on postoperative Day four. Patient serum creatinine decreased to 1.3 mg/dL. The patient was discharged on postoperative Day 10, sutures were removed on an outpatient basis on Day 14, and the Foley catheter was removed on postoperative Day 20. At short‐term follow‐up, the patient showed improvement in renal function and resolution of lower urinary tract symptoms. The patient is now under pulmonary consultation at another center and is planned for right PCNL after respiratory optimization.

## 3. Discussion

Giant bladder stone are rare and typically defined as stone > 10 cm in size or weighing > 100 g [6]. These stones present in a setting of prolonged neglected chronic obstruction, recurrent infection, urinary stasis, and poor health‐seeking behavior [7]. These giant stones can cause urinary obstruction and complications including recurrent urinary tract infections, obstructive uropathy, and hydronephrosis with renal impairment [8, 9].

X‐ray KUB, ultrasound (A + P), and CT (abdomen and pelvis) are usually done to determine the stone characteristics and for renal evaluation [10, 11]. Noncontrast CT scan abdomen and pelvis, high‐resolution CT chest and pulmonary function testing could not be performed because of financial constraints. Management of bladder stones is mostly dependent on their size, with transurethral and percutaneous approaches mostly preferred for smaller stones; open cystolithotomy remains the treatment of choice for large stones [12–14].

This case defines the challenges in managing neglected giant bladder stones with bilateral hydronephrosis and deranged renal function in elderly, medically complex patients with a collapsed left lung from resource‐limited settings. The unusual aspect of this case lies in the size of the bladder stone and the necessity to perform open cystolithotomy under local anesthesia with mild sedation due to underlying severe pulmonary compromise. The likely etiology of the bladder stone in this case includes chronic urinary stasis, recurrent infection, dehydration, and delayed healthcare access rather than significant outlet obstruction. Chest x‐ray demonstrated findings suggestive of left‐sided lung collapse. The exact etiology could not be definitely established because CT chest and pulmonary function testing were not available. Possible causes include chronic atelectasis, likely secondary to long‐standing smoking and infection. Lung collapse dramatically limits respiratory reserve, and general anesthesia with ventilation or spinal anesthesia because of sympathetic blockade and respiratory muscle impairment in a patient with markedly reduced pulmonary reserve could have precipitated respiratory failure. After multidisciplinary discussion, local infiltration with carefully titrated conscious sedation was considered the safest available option.

Local anesthesia is rarely used for open bladder procedures due to concerns regarding pain control and patient compliance. Despite the use of local anesthesia, the procedure was well tolerated with adequate analgesia and mild sedation. Given the patient′s severe pulmonary compromise and lack of safer anesthetic alternatives, this approach was considered both clinically justified and humane.

Preoperative and postoperative rehabilitation, including treatment of infection, postoperative pain management, continued chest physiotherapy, early mobilization, and good nursing care, was vital for the early recovery of our patient. This case emphasizes the versatility in adapting surgical and anesthetic approaches when general or regional anesthesia is unsafe; however, the risk–benefit ratio must be carefully weighed, highlighting the need for protocols in such settings. Furthermore, such cases emphasize that neglected urological disease still occurs in our healthcare settings and requires vigilance and early intervention.

This case report has several limitations. Noncontrast CT of the abdomen and pelvis, high‐resolution CT chest, and pulmonary function testing could not be performed because of financial constraints, which limited definitive characterization of both the renal/bladder pathology and the etiology of the lung collapse. Stone analysis and metabolic evaluation were not performed owing to the unavailability of these investigations at our government hospital. Long‐term follow‐up data, including the outcome of the planned right‐sided PCNL and eventual pulmonary status, are not yet available.

## 4. Conclusion

Open cystolithotomy under local anesthesia with mild sedation can be a safe, effective, and life‐saving alternative in patients with giant bladder stones with high anesthetic risk. This case highlights the importance of individualized patient care, multidisciplinary coordination, and surgical adaptability in low‐resource, high‐risk situations. It also emphasizes the ongoing need for improved healthcare access and screening in remote parts of Nepal to prevent such advanced disease presentations.

## Author Contributions

Milan Gyawali: conceptualization, literature review, surgical planning, manuscript drafting, and final approval. Ritamvara Oli: literature review, manuscript preparation, and critical revision. Sunil Regmi: surgical assistance, patient management, clinical data acquisition, and manuscript review. Purushottam Parajuli: radiological interpretation, diagnostic evaluation, and manuscript review. Akash Chitrakar: surgical assistance, manuscript revision. Rabindra Tamang: surgical assistance, literature review and critical revision.

## Funding

No funding was received for this manuscript.

## Disclosure

All authors have read and approved the final version of the manuscript. Dr. Milan Gyawali had full access to all data and takes responsibility for integrity and accuracy.

## Consent

Written informed consent for the procedure and for publication was obtained.

## Conflicts of Interest

The authors declare no conflict of interest.

## Data Availability

Data sharing not applicable to this article as no datasets were generated or analysed during the current study.

## References

[1] Schwartz B. F. and Stoller M. L. , The Vesical Calculus, Urologic Clinics of North America. (2000) 27, no. 2, 333–346, 10.1016/S0094-0143(05)70262-7. 10778475

[2] Aliabadi H. , Cass A. S. , Gleich P. , and Johnson C. F. , Vesical Calculi: Analysis of 89 Cases, Urology. (1985) 25, no. 1, 38–41.

[3] Ord J. , Khan A. , and Browning A. J. , Giant Bladder Stones: A Case Report and Review of the Literature, International Urology and Nephrology. (2003) 35, no. 3, 409–411.

[4] Koseoglu E. , Aslan G. , Sen B. H. , and Tuna B. , Giant Bladder Stone: A Rare Cause of Renal Failure, International Urology and Nephrology. (2006) 38, no. 2, 343–345.

[5] Türk C. , Neisius A. , Petřík A. , Seitz C. , Skolarikos A. , Thomas K. , Somani B. , Tailly T. , Gambaro G. , Bezuidenhout C. , and Lombardo R. , EAU Guidelines on Urolithiasis, 2025, European Association of Urology.

[6] Al-Ani A. , Al-Haddad M. , and Al-Saadi M. , Giant Bladder Stone: A Rare Case Report and Review of Literature, World Journal of Advanced Research and Reviews. (2025) 25, no. 1, 235–239.

[7] Acharya G. B. , Baral S. , Bijukchhe S. M. , Mishra S. , Bhusal K. , and Poudel S. , Giant Urinary Bladder Stone: A Rare Case Report, International Journal of Surgery Case Reports. (2024) 122, 110174, 10.1016/j.ijscr.2024.110174, 39146668. PMC11379588 39146668

[8] Hu J. , Phan A. T. , and Craig D. , A Rare Case of a Giant Bladder Stone Associated With Post-Obstructive Renal Failure Managed by Open Cystolithotomy, Cureus. (2023) 15, no. 5, 10.7759/cureus.39718, 37398827. PMC10309654 37398827

[9] Pires J. C. C. , de Brito B. B. , de Melo F. F. , Souza C. L. , and Oliveira M. V. , Giant Urinary Bladder Stone in a Middle-Aged Male, EMJ Urology. (2022) 10.33590/emjurol/21-00239.

[10] Ng C. and Tsung J. W. , Avoiding Computed Tomography Scans by Using Point-Of-Care Ultrasound When Evaluating Suspected Pediatric Renal Colic, Emergency Medicine. (2015) 49, no. 2, 165–171, 10.1016/j.jemermed.2015.01.017, 25934378. 25934378

[11] Bourcier J.-E. , Gallard E. , Redonnet J.-P. , Abillard M. , Billaut Q. , Fauque L. , Jouanolou A. , and Garnier D. , Ultrasound at The Patient′s Bedside for the Diagnosis and Prognostication of a Renal Colic, Ultrasound Journal. (2021) 13, no. 1, 10.1186/s13089-021-00246-2, 34807330. PMC8608963 34807330

[12] Katsimperis S. , Pikramenos K. , Livadas K. , Chatzikrachtis N. , and Bellos T. T. , Giant Bladder Stone: A Case Report, Cureus. (2022) 14, e25439, 10.7759/cureus.25439. PMC9239294 35774646

[13] Leslie S. W. , Sajjad H. , and Murphy P. B. , Bladder Stones, 2025, StatPearls Publishing. 28722973

[14] Assimos D. , Krambeck A. , Miller N. L. , Monga M. , Murad M. H. , Nelson C. P. , Pace K. T. , Pais V. M. , Pearle M. S. , Preminger G. M. , Razvi H. , Shah O. , and Matlaga B. R. , Surgical Management of Stones: American Urological Association/Endourological Society Guideline, Part I, Journal of Urology. (2016) 196, no. 4, 1153–1160, 10.1016/j.juro.2016.05.090, 27238616. 27238616

